# Associations of adenovirus-reactive immunoglobulins with atrial fibrillation and body mass index

**DOI:** 10.3389/fcvm.2023.1190051

**Published:** 2023-05-24

**Authors:** Nadezhda G. Gumanova, Polina D. Zlobina, Natalya L. Bogdanova, Hakob A. Brutyan, Elena N. Kalemberg, Victoria A. Metelskaya, Karapet V. Davtyan, Oksana M. Drapkina

**Affiliations:** Department of Biochemistry, National Research Center for Preventive Medicine (NRCPM), Moscow, Russia

**Keywords:** antibody microarray, atrial fibrillation, obesity, AdV-IgG, serum

## Abstract

Adenovirus (AdV) has been suggested to be involved in pathogenesis of atrial fibrillation (AF). We aimed to evaluate an association between AdV-specific immunoglobulins G in the serum (AdV-IgG) and AF. The present case-control study comprised two cohorts, including cohort 1 of patients with AF and cohort 2 of asymptomatic subjects. Initially, two groups, MA and MB, were selected from the cohorts 1 and 2, respectively, for serum proteome profiling using an antibody microarray to identify possible relevant protein targets. The data of microarray analysis indicated a possible overall increase in the total adenovirus signals in the group MA vs. group MB, suggesting potential relevance of adenoviral infection to AF. Then, the groups A (with AF) and B (control) were selected from the cohorts 1 and 2, respectively, to assay the presence and levels of AdV-IgG- by ELSA. The prevalence of AdV-IgG-positive status demonstrated a 2-fold increase in the group A (AF) compared with that in the group B (asymptomatic subjects); odds ratio 2.06 (95%CI: 1.11–3.84; *P* = 0.02). The prevalence of obesity demonstrated an approximately 3-fold increase in AdV-IgG-positive patients of the group A compared with that in AdV-IgG-negative patients of the same group A (odds ratio 2.7; 95% CI: 1.02–7.1; *P* = 0.04). Thus, AdV-IgG-positive reactivity was independently associated with AF, and AF was independently associated with BMI, indicating that adenoviral infection may be a possible etiological factor for AF.

## Introduction

Adenovirus (AdV) is a double-stranded DNA virus that causes respiratory, enteric, and conjunctival diseases in humans. Adenoviruses are nonenveloped virions 70–90 nm in diameter, with a capsid consisting of three main exposed structural proteins (hexon, fiber, and penton base) (1). Human adenoviruses are grouped into seven major species (A through G), including a total of 88 unique types with multiple genotypes (for example, Ad7a and Ad7d2). However, the nomenclature and classification systems are being revised, and various serotypes are associated with certain modes of infection (2). Adenovirus can be isolated from myocardial biopsies of patients with nonischemic cardiomyopathy. Adenovirus interferes with connexins junctions thus altering the cell-to-cell communications or electrical coupling of myocytes. Uncoupling of gap junctions leads to chaotic electrical activity in the atria or ventricles of the heart (3). Atrial fibrillation (AF) is characterized by irregular fibrillatory waves, and AF causes substantial morbidity due to elevated risk of stroke. Adenoviruses have been suggested to contribute to pathogenesis of AF, which is known to be caused by multiple factors. The present study was aimed to evaluate a potential role of adenoviruses as factors linked to AF by estimating the associations between adenovirus-specific immunoglobulins G (AdV-IgG) in the serum and AF.

## Materials and methods

### Subjects

The present study included two cohorts of subjects: cohort 1 comprised patients with AF (*N* = 98) and cohort 2 comprised asymptomatic subjects (*N* = 208).

**Cohort 1** with AF included patients treated in the National Research Center for Preventive Medicine (*N*RCPM), Ministry of Healthcare of Russian Federation, Moscow, Russia. These patients underwent primary pulmonary vein cryoballoon ablation using a 28-mm cryoballoon (Arctic Front Advance, Medtronic, USA) with simultaneous installation of an electrocardiogram (ECG) loop recorder (Reveal Linq, Medtronic, USA and SJM Confirm, Abbott, USA) from April 2017 to December 2022. All patients were over 18 years of age and suffered from symptomatic AF, with at least A2b score according to the European Heart Rhythm Association (EHRA) symptom classification for AF (4) and paroxysmal/persistent AF. The study has been registered at the ClinicalTrials.gov website [registration number NCT05170607; an overview of the protocol has been provided in our previous publication (5)]. The study protocol was compliant with the Declaration of Helsinki and WHO guidelines and was approved by the Independent Ethics Committee of NRCPM (number 01-06/17; February 2, 2017).

The follow up period of 12 months included scheduled visits at 3, 6, and 12 months after the ablation procedure to monitor and discontinue an antiarrhythmic therapy (excluding beta-blockers) after 3 months as appropriate. During the visits, the data of an implanted ECG loop recorder were retrieved and assessed. Patients with recurrent AF were subjected to an invasive electrophysiological examination to identify a possible cause of the condition. Based on the results of this examination, the patients were subjected to repeated radiofrequency ablation (RFA). If the lesions of the orifices of the pulmonary vein were detected, the observation continued for 12 months with the visits 3, 6, and 12 months after the procedure. The main goal of the follow up visits was to determine whether the treatment was effective. Follow up assessment included physical examination, 12-lead ECG, and retrieval and detailed analysis of information from the ECG loop recorders. Patient medical history, complaints, and the usage of antiarrhythmic drugs were considered. Patients had pulmonary vein-associated AF and AF (substrate AF) persisting after pulmonary vein isolation during the first or repeated procedures, with AF recurrence after the first procedure but with isolated pulmonary vein at the beginning of the second AF ablation or with AF recurrence after repeated ablation. The body mass index (BMI) of over 30 kg/m^2^ was used as a borderline for obese status.

Inclusion criteria were as follows: age ≥18 years; drug-resistant (documented ineffective treatment with at least one antiarrhythmic drug of type I or type III, including β-blockers); and symptomatic (≥EHRA 2b class) paroxysmal/persistent AF with at least two episodes and at least one documented episode. Diagnosis was confirmed based on the guidelines for clinical practice by the European Society of Cardiology and Russian Society of Cardiology, which included a standard 12-lead ECG recording or a single-lead ECG tracing of >30 s showing heart rhythm with no discernible repeating *P* waves and irregular RR intervals (when atrioventricular conductivity was not impaired) (6).

The exclusion criteria were as follows: breast feeding or pregnancy; acute inflammation; severe comorbidities (decompensated hypo- and hyperthyroidism and acute decompensation, severe liver failure, and chronic kidney diseases requiring dialysis); the history of previous catheter-based or surgical AF treatment; intracardiac thrombosis; contraindications to oral anticoagulants or heparin; congestive heart failure stages III and IV according to the New York Heart Association classification; left ventricular ejection fraction <35%; chronic obstructive pulmonary disease with established pulmonary hypertension; atrioventricular block stage II (type 2) and block stage III; significant structural heart disease (anomalies in the development of pulmonary vein, clinically significant coronary artery disease, and valvular pathology, including the conditions after installation of prosthetic heart valves); transient ischemic attack of the brain or stroke <6 months before admission; unstable angina or myocardial infarction <3 months before admission; coronary angioplasty or mammarocoronary or coronary artery bypass grafting <6 months before admission; severe oncological diseases in the stage of intoxication; and heart transplant.

The groups MA and A were selected from cohort 1 for proteomic profiling using microarrays and for AdV-IgG ELISA, respectively.

**Cohort 2** of asymptomatic volunteers included subjects over 18 years of age. This cohort was characterized in a previous study that investigated microcirculation (7). The following exclusion criteria were applied: any acute inflammation including oral or dental inflammation; hematological diseases; any chronic diseases; mental illness; autoimmune diseases; any types of medication therapy; pregnancy; and lactation. All participants gave their written informed consent to participate in the study. The study protocol was compliant with the Declaration of Helsinki and WHO guidelines and was approved by the Independent Ethics Committee of NRCPM (number 01-01/17).

The groups MB and B were selected from cohort 2 for proteomic profiling using microarrays and for AdV-IgG ELISA, respectively.

### Study groups

**Figure 1:**
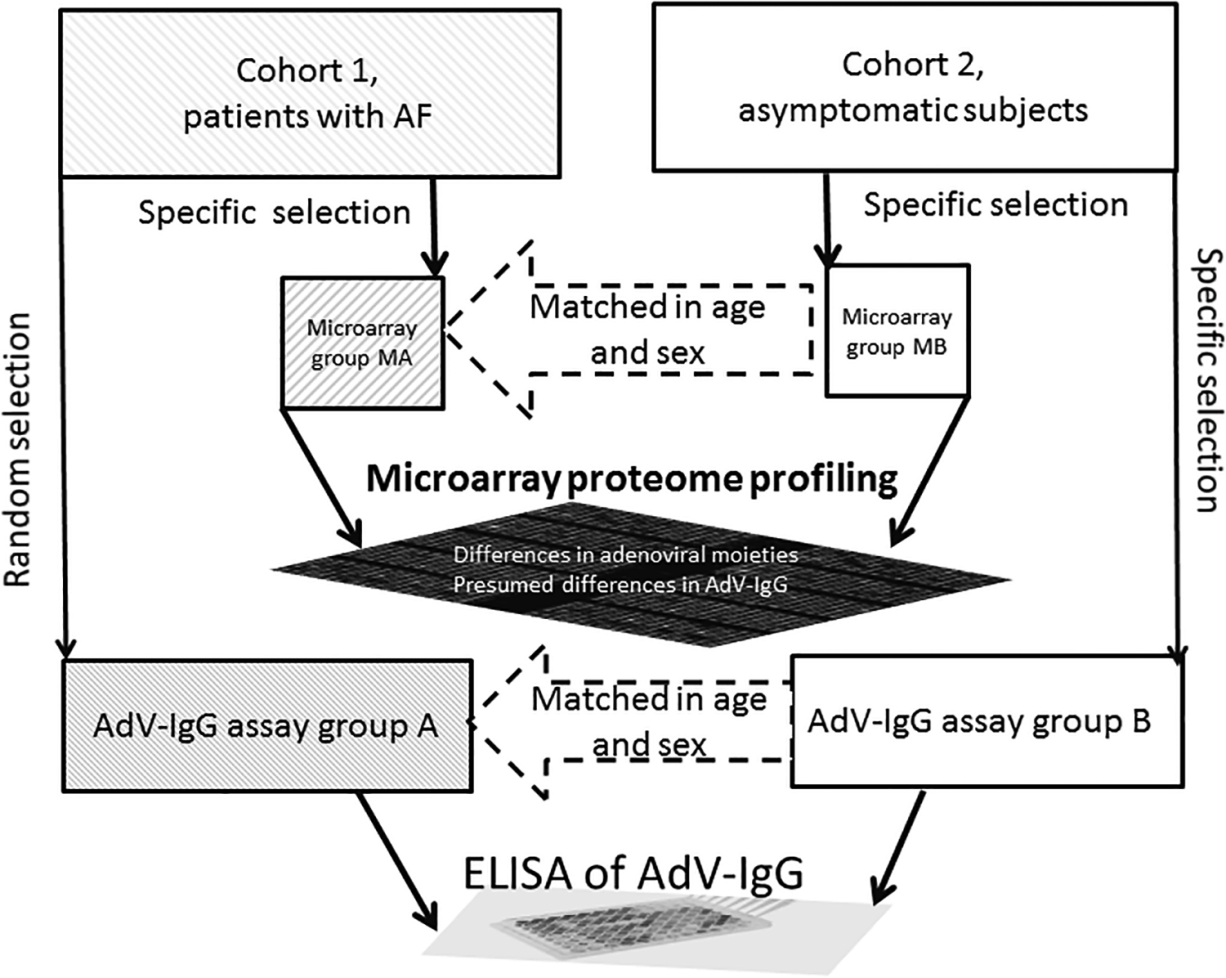
The workflow of analysis. Cohort 1 comprised patients with AF, and cohort 2 comprised asymptomatic subjects. The Microarray A (MA) and MB groups were selected from the cohorts 1 and 2, respectively, for target protein identification using antibody microarrays. The adenovirus-relevant targets (AdV-IgG) were then assays in the A (with AF) and B (asymptomatic subjects) groups using ELISA. The MB and B groups were selected to provide individual matches to the MA and A groups, respectively, to minimize variability due to sex and age.

The present case-control study included 4 groups: MA (case) and MB (control) for comparative proteomic profiling using antibody microarrays to identify potential targets of interest and groups A (case) and B (control) for specific evaluation of differential targets identified based on analysis of the data obtained by comparison of the group MA to group MB. Thus, the groups A and B were used to measure the levels of AdV-IgG by ELISA (Figure 1).

### Blood sampling

Blood was withdrawn from the cubital vein. The serum was obtained by centrifugation at 1,000 g for 15 min at 4°С. The serum and plasma were aliquoted and stored at −27°С. Serum aliquots were centrifuged at 10,000 g for 15 min at 4°С before proteomic analysis, and the protein concentrations were assayed by measuring the optical density (OD) at 260–280 nm using a NanoDrop One spectrophotometer (Thermo Scientific), using human serum albumin as the reference standard.

### Routine biochemical tests

Routine biochemical tests were performed using specific guidelines approved by Center for External Quality Control of Clinical Laboratory Testing of Russian Federation (www.fsvok.ru). C-reactive protein (CRP; mg/L) was assayed by high sensitivity quantitative immunoturbidimetric method enhanced with latex particles (universal range 0.3–350 mg/L; highly sensitive range 0.05–20 mg/L) using kits from DiaSys (Germany) by a Architect С 8000 analyzer (Abbot, USA). N-Terminal brain natriuretic peptide prohormone (NT-proBNP; pg/mL) was measured in the serum by chemiluminescent microparticle immunoassay (CV% < 10%) using an Radiometer AQT90 FLEX (Radiometer Medical ApS, Denmark).

### Antibody microarray analysis of labeled serum proteins

Serum proteins were diluted in phosphate buffered saline (PBS; Arrayit Corp.) to a concentration of 1 mg/ml and mixed with 20 µl of protein-labeling buffer. The proteins were labeled according to the manufacturer instructions by adding 1 µl of Green 540 reagent (Arrayit Corp.) for 60 min on ice to couple the dye to the proteins. The reaction was terminated by the addition of 10 µl of stop solution (Arrayit Corp.). After 30 min incubation on ice, inactivated unbound dye was removed by gel filtration through spin columns by centrifugation at 750 g for 2 min. Explorer antibody microarrays contained 656 antibodies per slide in two replicates for each antibody (ASB600, Full Moon Biosystems, USA; full list of antibodies is available on manufacturer's website at https://www.fullmoonbio.com/datasheets/ASB600_AbList.xls). The array slides were loaded with 500 ng of labeled proteins per slide and incubated with blocking buffer containing 3% dry milk (Full Moon Biosystems) for 1 h according to the manufacturer instructions. After the washing cycles, the microarrays were incubated with coupling buffer containing 3% dry milk (Full Moon Biosystems) for 2 h. After washing, the microarrays were dried by centrifugation (8, 9).

### Microarray slide scanning and image processing

Microarray slides were scanned using a laser scanner (InnoScan microarray scanner 900, Innopsys, France) using manual settings: scan mode: normal; velocity: 20 L/s; laser power: 5.0; photomultiplier gain: 100; pixel size: 10; and wavelength: 532 nm (8, 9). Gal-files and grid settings were loaded manually and matched with the positive controls. The images were analyzed using Mapix software version 7.0.0 (Innopsys, France), and labeled proteins were identified based on microchip-specific gal files and protein numbers corresponding to identifiers of the UniProtKB/Swiss-Prot database.

### AdV-IgG ELISA

Serum AdV-IgG concentrations were measured in the samples by an ELISA kit for human AdV-IgG (Fine Test, Wuhan Fine Biotech Co., Ltd., China) based on indirect ELISA. A 96-well plate was precoated with an AdV antigen. The samples were diluted 10-fold in sample dilution buffer and added to the wells, and the unbound proteins were washed with wash buffer. Then, a horse radish peroxidase (HRP)-conjugated secondary antibody was added to form an AdV-antigen/AdV-IgG/HRP-conjugate complex, which was detected using 3,3′,5,5′-tetramethylbenzidine HRP substrate. OD was read using Tecan Infinite 200 PRO microplate reader (Switzerland) at 450 nm with a reference wavelength at 620 nm, and the data were evaluated using Magellan software. The cutoff value corresponded to the mean OD of a negative control (approximately 0.05) plus 0.1. Samples with OD less than the cutoff value were considered negative for AdV-IgG, and the samples with OD higher than the cutoff value were considered positive for AdV-IgG.

### Statistical analysis

Statistical evaluation was preformed using SPSS (IBM). Continuous variables are presented as the mean  ± SD, and categorical variables are presented in percentages. Comparisons between sex, age, BMI, obesity, and C-reactive protein in the cohorts 1 and 2 were performed with Mann-Whitney U test. Associations between AF, obesity, and IgG-AdV status in the groups A with B were evaluated based on odds ratio (OR) with 95% confidential interval (95%CI). Multivariate multinomial logistic regression in combined groups A and B for various variables, including obesity, IgG-ADV status, and BMI, was assessed based on chi-square test with phi coefficient (Wald); 2-sided Fisher's exact test (with 95% CI) was used for analysis of the Exp(B) values.

The spot values on the images were expressed in median pixel intensity after the background at the referenced wavelength was subtracted. Parallel spots with high variability (CV over 25%) were withdrawn from subsequent analysis. The *P*-values <0.05 were considered significant.

## Results

### Characteristics of the groups

Cohort 1 with AF included 197 patients treated in NRCPM (Supplementary Table S1). All patients were diagnosed with AF. The mean age at the onset of arrhythmia was 53.24 ± 10.55 years, and the duration of AF history was 4.0 ± 2.7 years. The most frequent comorbidities included hypertension (75.63%) and obesity (53.2%). Diabetes mellitus type 2 was diagnosed in 28 patients (14.21%) based on fasting plasma glucose level ≥126 mg/dl (7.0 mmol/L), 2-h plasma glucose level ≥200 mg/dl (11.1 mmol/L) during a 75-g oral glucose tolerance test, and HbA1c ≥6.5% (48 mmol/mol) (Supplementary Table S1). The follow-up period has been completed for 187 patients, and 41 patients withdrew from the study due to non-compliance with the follow-up visits or rejection of reoperation. Remaining 146 patients with AF were classified as pulmonary vein-associated AF (*N* = 123) and substrate AF (*N* = 23).

Cohort 2 included a total of 208 asymptomatic subjects who underwent preventive counseling in NRCPM. General demographic, anthropometric, and biomarker characteristics of this cohort were not significantly different from the healthy reference values, with the following median values (25–75 percentiles): 55 years of age (from 48 to 60 years); body mass index 27 kg/m^2^ (from 24.5 to 29.7 kg/m^2^); systolic blood pressure 130 mm Hg (from 122 to 144 mm Hg); diastolic blood pressure 80 mm Hg (from 80 to 90 mm Hg); total cholesterol 6.0 mmol/L (from 5.3 to 6.9 mmol/L); triglycerides 1.2 mmol/L (from 0.9 to 1.7 mmol/L); low density lipoprotein cholesterol 4.3 mmol/L (from 3.6 to 5.1 mmol/L); high density lipoprotein cholesterol 1.0 mmol/L (from 0.8 to 1.2 mmol/L); apolipoprotein A1 150 mg/dl (from 135 to 170 mg/dl); glucose 5.1 mmol/L (from 4.7 to 5.5 mmol/L); insulin 8.1 µIU/ml (from 5.8 to 11.5 µIU/ml); homeostatic model assessment for insulin resistance (HOMA-IR) 1.8 (from 1.2 to 2.6); C-reactive protein 2.9 mg/L (from 1.8 to 4.5 mg/L); and creatinine 86 µmol/L (from 76 to 96 µmol/L). These parameters indicated that the participants of cohort 2 should be considered apparently healthy.

### Microarray proteome profiling in the MA and MB groups

The MA group included a total of 7 patients from the cohort 1 with AF (all women with pulmonary vein-associated AF (*N* = 4) and substrate AF (*N* = 3)). The matched control group MB included a total of 3 asymptomatic subjects (all women) from the cohort 2. These two groups were used for comparative microarray proteome profiling for identification of potential differential protein targets to minimize the impact of sex and age.

**Figure 2:**
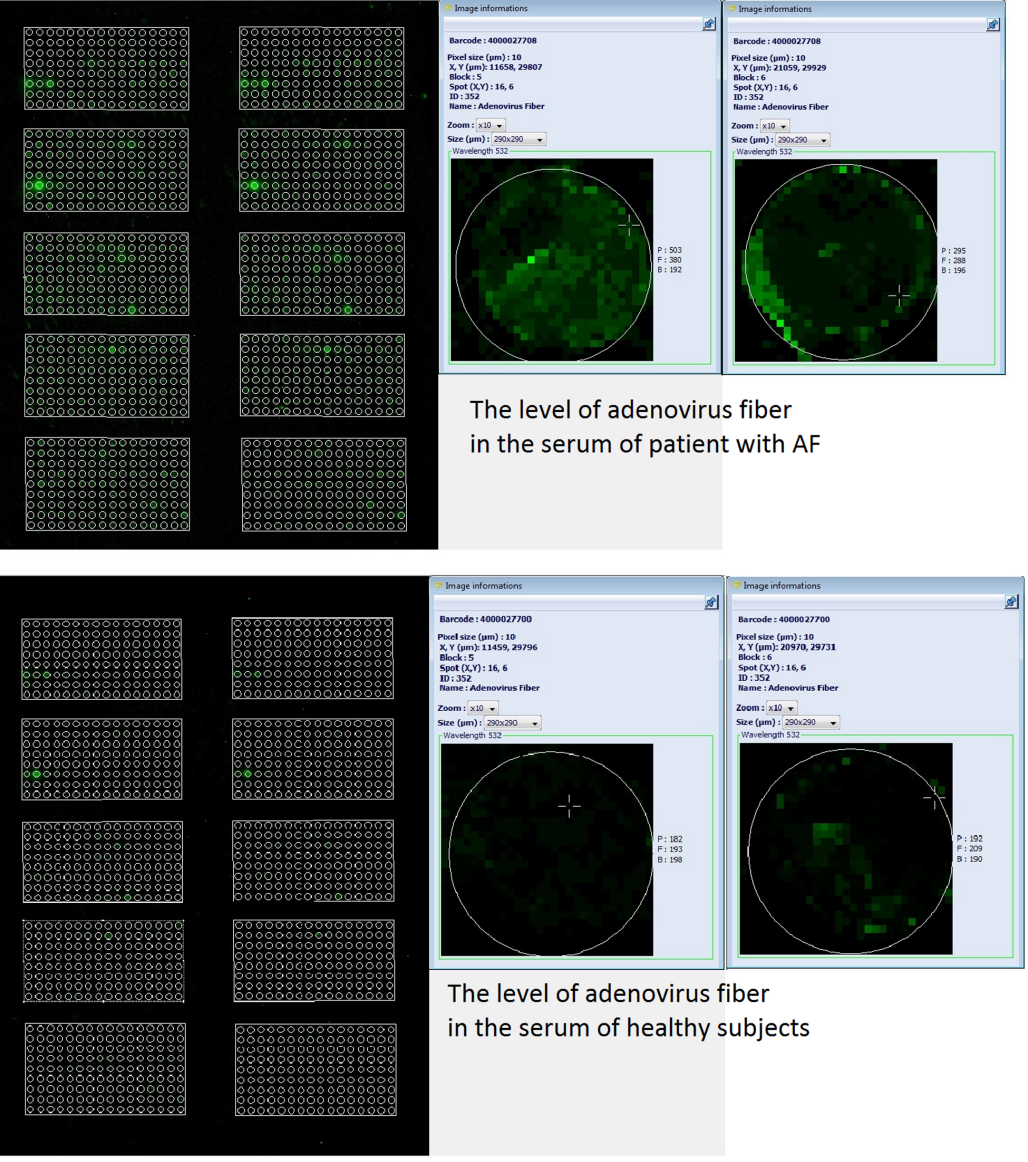
Images of two microarrays from a representative slide of Explorer antibody microarray (ASB600, Full Moon Biosystems, USA) with 656 antibodies per slide in two replicates for each antibody. Labeled serum proteins were loaded at 500 ng per slide. Scan settings: velocity: 20 L/s; laser power: 5.0; detector gain: 100; pixel size: 10; wavelength: 532 nm. Enlarged images of individual samples illustrate the differences in the level of adenovirus fiber in the serum of patient with AF (two images in parallel on top) vs. that in the serum of healthy subject (two images in parallel at the bottom), as labeled in the image.

Specific proteins were identified, quantified, and compared in the serum using Explorer antibody microarrays with 656 antibodies per slide in two replicates for each antibody. Explorer antibody microarray contained four various antibodies reactive toward the following adenovirus moieties: adenovirus (no UniProt number), adenovirus fiber (no UniProt number), adenovirus type 2 E1A (UniProt ID: P03254), and adenovirus type 5 (UniProt ID: P03255) (Supplementary Table S2). The total of the signals of all four adenoviral moieties was significantly different between the MA and MB groups. Notably, the levels of adenovirus fiber tended to increase in the MA group vs. the MB group (mean ± SD, pixel intensity minus background: 183 ± 43 vs. 155 ± 71, respectively; *P* = 0.067) (Supplementary Table S2 and Figure 2). Since adenovirus fiber is considered a clear indicator of adenoviral infection, anti-adenovirus IgGs were selected as the protein target, and the levels of AdV-IgG were assayed by ELISA kits.

### AdV-IgG assay

The A group (*N* = 91) was randomly selected from the cohort 1 with AF and the matching B group (*N* = 94) was selected from cohort 2 to match the age and sex (Supplementary Table S3). The prevalence of AdV-IgG-positive reactivity demonstrated a 2-fold increase in group A vs. group B, with the odds ratio (OR) of 2.06 (95%CI: 1.11–3.84; *P* = 0.02) (Supplementary Table S3). The prevalence of obesity was increased in AdV-IgG-positive patients of group A approximately 3-fold compared with that in AdV-IgG-negative patients of the same group (OR = 2.7; 95% CI: 1.02–7.1; *P* = 0.04) in contrast to the results obtained in the control group B (Supplementary Table S4). The data indicated the lack of significant associations between AdV-IgG status and arterial hypertension, diabetes mellitus type 2, coronary heart disease, stroke, or transient ischemic attack (Supplementary Table S1). Since these parameters were not associated with AdV-IgG status, they were not included as variables in multivariate logistic regression. The data of ROC analysis in combined groups A and B (*N* = 180) revealed the associations between AdV-IgG, BMI, obesity, and AF (Table 1). AdV-IgG-positive status was associated with AF (*P* = 0.048) and BMI (*P* = 0.033) (Table 1), and AF was strongly associated with BMI (*P* = 0.007). Since BMI, obesity, and AF have been associated with AdV-IgG status, these parameters were included in polynomial logistic regression. Arterial hypertension, diabetes mellitus type 2, coronary heart disease, stroke, or transient ischemic attack were not included because these parameters were not associated with AdV-IgG status according to the data of univariate ROC analysis. (Supplementary Table S1). Multivariate analysis in combined groups A and B was used to discriminate the explanatory variables by multinomial logistic regression (Table 2). The results indicated that AdV-IgG-positive status and BMI had an independent impact on AF (Table 2). Obesity status had a tendency (*P* = 0.074) for an association with AdV-IgG-positive status. No independent statistically significant impact of AdV-IgG-positive status on obesity was detected.

**Table 1:** Associations between AdV-IgG status, AF, obesity, and BMI in the combined A (AF) and B (asymptomatic) groups (*N* = 180) determined by univarite ROC-analysis.

Variables	AUC	*P*	Asymptotic 95% CI	
			Lower limit	Upper limit
*State variable: AdV-IgG status*				
BMI	0.596	0.033*	0.510	0.683
Obesity	0.581	0.073	0.495	0.667
AF	0.589	0.048*	0.502	0.676
*State variable: AF*				
BMI	0.617	0.007*	0.535	0.699
Obesity	0.579	0.068	0.495	0.662
IgG-ADV status	0.582	0.058	0.498	0.665
*State variable: Obesity*				
AF	0.581	0.064	0.496	0.666
IgG-ADV status	0.576	0.082	0.492	0.660

AUC, area under the curve.

*P < 0.05.

**Table 2:** Explanatory variable assessment for AF, obesity and BMI using multivariate multinomial logistic regression in combined groups A and B (*N* = 180).

Model	Variable	Wald test	*P*	95% CI for Exp(B)	
				Lower limit	Upper limit
*AF as dependent variable*					
Model 1	Obesity	3.325	0.068	0.958	3.262
	IgG-ADV status	3.969	0.046*	1.010	3.578
Model 2	BMI	5.576	0.018*	0.864	0.987
	IgG-ADV status	3.886	0.049*	1.004	3.577
*Obesity as dependent variable*					
Model 2	AF	3.325	0.068	0.307	1.044
	IgG-ADV status	3.182	0.074	0.943	3.484
*IgG-ADV status as dependent variable*					
Model 1	BMI	1.899	0.168	0.889	1.021
	AF	3.924	0.048*	1.007	3.578
Model 2	Obesity	3.182	0.074	0.943	3.484
	AF	3.969	0.046*	1.010	3.578

*P < 0.05.

The differences in the demographic, clinical, and biochemical parameters between AdV-IgG-positive and AdV-IgG-negative patients of group A were not detected, including similar levels of NT-proBNP and C-reactive protein (data not shown). The levels of AdV-IgG in the group A were not significantly different between patients with pulmonary vein-associated AF and substrate AF.

Thus, AdV-IgG-positive reactivity was independently associated with AF, and AF was independently associated with BMI, suggesting that adenoviral infection may be a possible etiological factor for atrial fibrillation.

## Discussion

We used the antibody microarray technology in a previous study for serum protein profiling of patients with coronary artery stenosis, and differential proteins have been validated using in-house ELISA (9).

Protein microarrays were developed in 1980s (10). Advanced microchips were then constructed, and the data attracted considerable interest for analysis of a large number of proteins in a minimal volume of samples from patients, including blood, serum, and other media (11, 12).

Certain microarray modifications enable the assays of up to 9,000 proteins and contain over 4,000 antibodies per slide. In general, antibody microarray technology is considered a useful tool for proteomic analysis and identification of novel biomarkers of various diseases and multiple alterations of the signaling pathways (13, 14). The present study used antibody microarrays for serum profiling of patients with AF by comparison with healthy subjects to identify differential targets. The results indicated that a total of four AdV moieties were increased in patients with AF (Table 2). Adenovirus fiber is one of three major proteins of adenovirus capsid, which also includes the hexon and penton base (15, 16). The fiber includes a C-terminal homotrimeric globular knob and an N-terminal elongated shaft, which mediate initial interactions with the receptors and anchoring of the fiber into the viral capsid, respectively (15, 16). Detection of adenovirus fiber in the serum is a clear indication of viral infection. Detection of IgGs against AdV in the serum by ELISA confirmed current or previously experienced viral infection (Figure 2). Typically, AdV infection is associated with mild respiratory manifestations. Acute inflammation is known to be associated with increased C-reactive protein levels and was thus excluded based on the exclusion criteria. Hence, we did not have patients with acute phase of inflammation, and this may be one of the reasons why there was the lack of associations between AdV-IgG status and C-reactive protein. Similarly, there was a lack of associations between NT-proBNP, which may be elevated in acute phase of inflammation, and IgG-AdV status, which is presumed to be present regardless of inflammatory status. However, the prevalence of AdV-IgG in patients with AF was 2-fold higher than that in asymptomatic subjects, suggesting that AdV infection is associated with AF. This result is consistent with known associations of adenoviruses with life-threatening diseases, including viral myocarditis that causes sudden cardiac death in 59% of patients (17, 18).

AdV infection has been considered as a possible etiological factor for atrial fibrillation, although the molecular mechanisms of infection-induced arrhythmogenesis are unknown (3). These mechanisms may be related to transcriptional and posttranslational modifications that disrupt the gap junctions. A reduction in the cellular coupling and ion channel functions induced by AdV infection was proposed to generate an arrhythmogenic substrate prior to a detectable immune response or the development of cardiomyopathy, indicating that electrical disturbances caused by an active infection may occur prior to an inflammatory response (3).

Obesity is linked to a number of chronic diseases and conditions, including chronic airway obstruction, pulmonary dysplasia, and cardiomyopathy (2, 19, 20). Additionally, a series of human and animal studies revealed associations of certain human AdV types with obesity (21). Infection by these viruses has been proposed as a possible cause of obesity epidemic (22). The data of the present study indicated that the prevalence of obese patients was approximately 3-fold increased in AdV-IgG-positive patients of the group A with AF compared with that in AdV-IgG-negative patients of the same group, and these differences were not observed in the control group B (Supplementary Table S4). This finding is consistent with the results of the case-control and cohort studies, which demonstrated that overall prevalence of human AdV-36 infection in obese population was 32% in the case group and 27% in the control group (22). These effects of Adv36 infection were suggested to be related to insulin-independent glucose uptake (23). Adv36 upregulates phosphoinositide 3-kinase (PI3K) signaling via Ras, increasing cellular glucose uptake by glucose transporters Glut1 and Glut4 despite downregulation of insulin receptor substrate signaling (23). Oxidative stress represents another possible mechanism of the development of obesity, although the effects of adenovirus in this context are unknown (21). Additionally, the results of a Framingham study and meta-analysis indicated that a rise in BMI parallels a marked increase in AF risk (24, 25). AF incidence is reduced by 7% for every 1 kg/m^2^ drop in BMI (26). These associations between obesity and AF risk are often attributed to various coexisting cardiovascular risk factors linked to obesity, including diabetes and hypertension (27).

The levels of AdV-IgG in the case group A were not significantly different between patients with pulmonary vein-associated AF and substrate AF apparently because resistance to treatment was not associated with previous adenovirus infection.

## Limitations

The present study has a number of limitations. The small number of patients in the microarray analysis groups was due to financial constraints, as the cost of microarrays is relatively high.

Ideally the control cohort may be randomly selected to have cardiovascular risks similar to those of the case group, while having no AF.

## Conclusions

AdV-IgG-positive status of patients was independently associated with AF, and AF was independently associated with BMI. Thus, adenoviral infection may be a possible etiological factor for atrial fibrillation.

## Data Availability

'The original contributions presented in the study are included in the article/**Supplementary Materials**, further inquiries can be directed to the corresponding author.
