# Supplementary material for: Associations of adenovirus-reactive immunoglobulins with atrial fibrillation and body mass index
Source: Front Cardiovasc Med. 2023 May 24;10:1190051. doi: 10.3389/fcvm.2023.1190051 (PMC10246773; doi:10.3389/fcvm.2023.1190051)
Supplement: Supplementary file 1 [file Table1.docx]

Table 1S. Clinical and biochemical characteristics of subjects of cohorts 1 (with AF; N = 197) and 2 (asymptomatic; N = 208) and associations between cardiovascular risk factors with AdV-IgG status.

| Parameter | Cohort 1 | | AdV-IgG status associations in cohort1, according to ROC-analysis | | Cohort 2 | |  |  | |  |
| --- | --- | --- | --- | --- | --- | --- | --- | --- | --- | --- |
|  | Mean/  value | SD | AUC (95% CI); P | | | Mean/value | SD | |  | |
| Sex (women, %) | 42.1 | - | >0.05 |  | | 40.8 | - | |  | |
| Age (years) | 57.8 | 10.5 | >0.05 |  | | 55.0 | 11.2 | |  | |
| BMI (kg/m^2^) | 30.50 | 4.63 | 0.62(0.48-0.75); 0.075 | | | 27.0* | 3.5 | |  | |
| Obesity (BMI > 30 kg/m^2^, %) | 53.2 | - | 0.62(0.49-0.74); 0.078 | | | 24.5 | - | |  | |
| Age at the onset of arrhythmia (years) | 53.24 | 10.55 | NA |  | | - | - | |  | |
| Arterial hypertension (%) | 75.63 | - | >0.05 |  | | - | - | |  | |
| Diabetes mellitus type 2 (%) | 14.2 | - | >0.05 |  | | - | - | |  | |
| Coronary heart disease (%) | 7.6 | - | >0.05 |  | | - | - | |  | |
| Stroke or transient ischemic attack in anamnesis (%) | 11.6 | - | >0.05 |  | | - | - | |  | |
| Left atrial diameter (mm) | 42.91 | 5.04 |  |  | | - | - | |  | |
| Left ventricular ejection fraction (%) | 61.36 | 8.04 | >0.05 |  | | - | - | |  | |
| Left ventricular end-diastolic diameter (mm) | 51.92 | 6.39 | >0.05 |  | | - | - | |  | |
| Left ventricular hypertrophy (wall thickness >14 mm, %) | 8.63 | - | >0.05 |  | | - | - | |  | |
| NT-proBNP (pg/mL) | 284.3 | 482.9 | >0.05 |  | | - | - | |  | |
| C-reactive protein (mg/L) | 6.7 | 10.2 | >0.05 |  | | 2.9 | 1.5 | |  | |

*P<0.05 versus cohort 1.

NA-not applicable

Table 2S. Protein targets in the MA (with AF) and MB (asymptomatic) groups according to the results of microarray proteome profiling.

| Name according to ASB600 | ID ASB600 | ID UniProt | Signal intensity in the MA and MB groups (pixels; mean ± SD) | | |
| --- | --- | --- | --- | --- | --- |
|  |  |  | Group MA AF; N = 7 | Group MB asymptomatic; N = 3 | P |
| Adenovirus | 369 | NA | 154±77 | 196±105 | 0.524 |
| Adenovirus fiber | 352 | NA | 183±43 | 155±71 | 0.067 |
| Adenovirus type 2 E1A | 243 | P03254 E1A_ADE02 | 114±93 | 247±163 | 0.198 |
| Adenovirus type 5 E1A | 242 | P03255 E1A_ADE05 | 69±69 | 131±93 | 0.439 |
| Total signals of adenovirus moieties |  |  | 182±115 | 129±72 | 0.033* |

*P<0.05 according to Mann-Whitney test.

NA - not applicable

Table 3S. IgG-AdV status in the groups A (patients with AF) and B (asymptomatic subjects).

| Groups, N | AdV-IgG-positive, N (%) | AdV-IgG-negative, N (%) | OR (95%CI); P |
| --- | --- | --- | --- |
| A, 91 | 66 (73) | 25 (27) | 2.06 (1.11-3.84); 0.02 |
| B, 89 | 50 (56) | 39 (44) |  |

OR, odds ratio.

Table 4S. Associations between obesity and IgG-AdV status in the groups A (patients with AF) and B (asymptomatic subjects).

| Groups, IgG-AdV status (N) | Obesity, N (%) | BMI < 30 kg/m^2^, N (%) | OR (95%CI); | P |
| --- | --- | --- | --- | --- |
| *Group A (91)* |  |  |  |  |
| AdV-IgG-negative (25) | 8 (32) | 17 (68) | 2.7 (1.02-7.1) | 0.04 |
| AdV-IgG-positive (66) | 37 (56) | 29 (44) |  |  |
| *Group B (89)* |  |  |  |  |
| AdV-IgG-negative (39) | 12 (31) | 27 (88) | 1.3 (0.51-3.01) | 0.6 |
| AdV-IgG-positive (50) | 18 (20) | 32 (64) |  |  |
